# Characterisation of tumour vasculature in mouse brain by USPIO contrast-enhanced MRI

**DOI:** 10.1038/sj.bjc.6604389

**Published:** 2008-05-27

**Authors:** G Gambarota, W Leenders, C Maass, P Wesseling, B van der Kogel, O van Tellingen, A Heerschap

**Affiliations:** 1 Department of Radiology, Radboud University Nijmegen Medical Center, Nijmegen, The Netherlands; 2 Department of Pathology, Radboud University Nijmegen Medical Center, Nijmegen, The Netherlands; 3 Department of Radiotherapy, Radboud University Nijmegen Medical Center, Nijmegen, The Netherlands; 4 Department of Pharmacology, Dutch Cancer Institute, Amsterdam, The Netherlands

**Keywords:** mouse brain, high-field MR, Gd-DTPA, USPIO, VEGF, contrast agent, tumour

## Abstract

To enhance the success rate of antiangiogenic therapies in the clinic, it is crucial to identify parameters for tumour angiogenesis that can predict response to these therapies. In brain tumours, one such parameter is vascular leakage, which is a response to tumour-derived vascular endothelial growth factor-A and can be measured by Gadolinium-DTPA (Gd-DTPA)-enhanced magnetic resonance imaging (MRI). However, as vascular permeability and angiogenesis are not strictly coupled, tumour blood volume may be another potentially important parameter. In this study, contrast-enhanced MR imaging was performed in three orthotopic mouse models for human brain tumours (angiogenic melanoma metastases and E34 and U87 human glioma xenografts) using both Gd-DTPA to detect vascular leakage and ultrasmall iron oxide particles (USPIO) to measure blood volume. Pixel-by-pixel maps of the enhancement in the transverse relaxation rates (Δ*R*_2_ and Δ*R*_2_^*^) after injection of USPIO provided an index proportional to the blood volume of the microvasculature and macrovasculature, respectively, for each tumour. The melanoma metastases were characterised by a blood volume and vessel leakage higher than both glioma xenografts. The U87 glioblastoma xenografts displayed higher permeability and blood volume in the rim than in the core. The E34 glioma xenografts were characterised by a relatively high blood volume, accompanied by only a moderate blood–brain barrier disruption. Delineation of the tumour was best assessed on post-USPIO gradient-echo images. These findings suggest that contrast-enhanced MR imaging using USPIOs and, in particular, Δ*R*_2_ and Δ*R*_2_^*^ quantitation, provides important additional information about tumour vasculature.

Growth and metastasis of solid tumours are considered to strictly depend on angiogenesis (Folkman, 1992). However, especially in organs with high vessel densities such as lung, liver and brain, the dependency of infiltrative tumours on angiogenesis is not always evident because the pre-existent vasculature may supply sufficient blood to the tumour cells to allow tumour progression. In the brain, the prototypes of such tumours are the diffuse infiltrative gliomas. Importantly, co-opted blood vessels in such brain tumours may be relatively unaffected by the tumour cells and may retain the highly specialised blood–brain barrier function (Kusters *et al*, 2002; Leenders *et al*, 2003). This probably explains why diffuse infiltrative parts of glioblastoma multiforma, the most frequent and most malignant glial brain tumour, as well as low-grade, infiltrative gliomas do not show enhancement in Gadolinium-DTPA (Gd-DTPA) magnetic resonance imaging (MRI) (Leenders *et al*, 2003). Indeed, in brain tumours, Gd-DTPA leaks from the vascular system into the interstitial space only when the blood–brain barrier is disrupted, that is, when the tumour increases the permeability of pre-existing blood vessels or induces the growth of leaky blood vessels.

The detailed characterisation of tumour vasculature with respect to permeability and vascular volume provides essential insight into tumour physiology and is a prerequisite to investigate and evaluate tumour response to antiangiogenic therapy. Apart from Gd-DTPA, superparamagnetic ultrasmall iron oxide particles (USPIO)-enhanced MRI has been demonstrated to be useful for the characterisation of the tumour vasculature (Dennie *et al*, 1998; Su *et al*, 1998; Le Duc *et al*, 1999; Tropres *et al*, 2001; Turetschek *et al*, 2001; Kusters *et al*, 2002; Bremer *et al*, 2003; Leenders *et al*, 2003; Pathak *et al*, 2003; Robinson *et al*, 2003; van Laarhoven *et al*, 2006). Although Gd-DTPA is used routinely in the clinic since many years, USPIOs have only recently been introduced for human applications (Enochs *et al*, 1999; Harisinghani *et al*, 2003). Superparamagnetic ultrasmall iron oxide particle contrast agents remain intravascular for a prolonged period of time and drastically increase the transverse water proton MR relaxation rate, the USPIO T2 relaxivity being up to 20 times that of Gd-DTPA (Gambarota *et al*, 2006). Superparamagnetic ultrasmall iron oxide particle thus provides a good marker for alterations in blood volume in different tissues, a characteristic that might be exploited for tumour detection and characterisation, such as in human brain (Enochs *et al*, 1999; Varallyay *et al*, 2002; Taschner *et al*, 2005). Although the USPIO-induced signal dephasing extends beyond the size of the main magnetic field perturbers, that is, the blood vessels, this does not seem to confound tumour delineation at the human scale. Previous studies have already shown that magnetic susceptibility effects caused by USPIO can be used to assess relative blood volumes within tumours (Dennie *et al*, 1998; Le Duc *et al*, 1999). In particular, the change in the transverse relaxation rates Δ*R*_2_ and Δ*R*_2_^*^ after injection of USPIO provides an index proportional to the blood volume of the microvasculature and macrovasculature, respectively (Kennan *et al*, 1994; Weisskoff *et al*, 1994). In murine brain tumours, however, so far only qualitative measurements of the decrease in signal intensity following USPIO administration have been reported (Leenders *et al*, 2003).

We have previously shown that treatment of angiogenic brain tumours with ZD6474, a tyrosine kinase inhibitor with specificity towards vascular endothelial growth factor (VEGF) receptor 2 and the epidermal growth factor receptor, resulted in conversion to a nonangiogenic phenotype, characterised by perivascular growth along pre-existent vessels (Leenders *et al*, 2004). Importantly, this treatment also resulted in restoration of the blood–brain barrier and a concomitant inability to detect these tumours with conventional Gd-DTPA MRI. This restoration of the blood–brain barrier may thus lead to overestimation of therapeutic efficacy. To detect infiltratively growing brain tumours, MR-based quantification of blood volume may have added value in evaluating antiangiogenic therapies. Indeed, we have previously shown that intracerebral lesions of Mel57, a human melanoma cell line with no notable expression of VEGF-A, were not detectable in Gd-DTPA MR images, yet were visible in T2 images after intravenous injection of USPIOs (Leenders *et al*, 2003). Similar results have been obtained with intracerebral U87 xenografts (Claes *et al*, 2008). This detection was based on the low intratumoral vascular volume, relative to the surrounding brain parenchyma.

In the current study, we aimed to develop imaging protocols that better define the vascular characteristics of different tumours. Tumour models were used, of which the vasculature has been extensively characterised at the morphological level. Tumour-bearing mice were subjected to both Gd-DTPA and USPIO imaging. Blood volume maps were generated for all tumours, by calculating Δ*R*2 and Δ*R*2^*^ following USPIO administration.

## MATERIALS AND METHODS

### Tumour models

E34 is a transplantable human glioblastoma xenograft line that is maintained as subcutaneous tumours in mice (Claes *et al*, 2008 accepted in Brain Pathology). On the day of intracerebral tumour injection, a nude mouse carrying a subcutaneous E34 xenograft was killed. The tumour was removed under sterile conditions and minced to small pieces using a scalpel. A tumour cell suspension was prepared by gently filtering the homogenate through a 70-*μ*m mesh filter. Twenty microlitres of this cell suspension was transcranially injected, 3 mm under the skull under standardised conditions. The procedure was carried out with 7- to 8-week-old Balb/c nude mice under anaesthesia with a mixture of 1.3% isoflurane in N_2_O/O_2_. This procedure reproducibly results in neurological symptoms due to intracerebral tumour growth 24–30 days after injection.

U87 glioma cells and stable VEGF-A_165_ transfectants of Mel57 cells were cultured in Dulbecco's modified essential medium supplemented with 10% fetal calf serum essentially as described before (Kusters *et al*, 2002). For the U87 tumour model, 1 × 10^5^ cells were injected transcranially using the same procedure as described for E34. Mel57-VEGF-A_165_ cells were injected in the internal carotid artery using a microsurgical procedure that was described before (Kusters *et al*, 2001). Using this melanoma metastasis model, multiple lesions develop in the brain parenchyma that display all hallmarks of angiogenesis (including leaky, highly dilated vessels). All experiments were approved by the Animal Experimental Committee of the Radboud University Nijmegen.

### Magnetic resonance imaging

Magnetic resonance imaging was performed as described in a previous study (Leenders *et al*, 2003). Mice were anaesthesised with isoflurane (1.5–2%) in N_2_O/O_2_; in each mouse, a catheter was inserted in a lateral tail vein to allow administration of contrast agent. A 10-mm diameter surface transmitter/receiver RF coil was positioned over the mouse head. During the measurements, the body temperature of the mice was maintained with a warm water pad.

Magnetic resonance imaging experiments were performed on a 7 T/200 mm horizontal-bore MR magnet interfaced to an SMIS console and equipped with a gradient insert (gradient strength=150 mT m^−1^ and rise time=150 *μ*s). The image acquisition protocol started with three gradient-echo scout images for slice positioning. The protocol of the Gd-DTPA contrast-enhanced MRI consisted of T1-weighted multislice gradient-echo images with TR/TE=400/6 ms, voxel size=136 × 136 × 1000 *μ*m, 16 slices, receiver bandwidth=100 kHz, scan time=1 min 16 s. The images were acquired prior to and 1, 2, 10 and 20 min after administration of Gd-DTPA (Magnevist^®^; Schering, Berlin, Germany) at a dose of 0.2 mmol kg^−1^.

A number of pilot experiments were performed to optimise the protocol of blood volume measurements. At the dose of USPIO agent used in the current study, a good compromise between signal-to-noise ratio (SNR) and contrast enhancement was found for values of TE in the range of 5–10 ms. As susceptibility effects scale linearly with the static magnetic field strength, longer (shorter) values of TE might be advantageous for blood volume measurements at a lower (higher) field. The protocol of the USPIO contrast-enhanced MRI consisted of multislice gradient-echo (TR/TE=1500/7 ms, 16 slices, voxel size=136 × 136 × 1000 *μ*m, receiver bandwith=100 kHz, scan time=4 min 54 s) and spin-echo (TR/TE=2000/9 ms, 16 slices, voxel size=136 × 136 × 1000 *μ*m, receiver bandwith=100 kHz, scan time=6 min 32 s) imaging performed prior to and following administration of a USPIO blood-pool agent (Sinerem; Guerbet, Roissy, France; 170 *μ*g Fe/mouse). Magnetic resonance imaging with USPIO was performed 2–3 h after Gd-DTPA imaging in mice with intracerebral Mel57-VEGF-A_165_ (*n*=4), E34 (*n*=3) or U87 (*n*=4) tumours. This time gap allowed complete wash out of Gd-DTPA and enabled a correlation between Gd-DTPA and USPIO enhancement.

### Data analysis

For each animal, pixel-by-pixel Δ*R*2^*^ maps were obtained from the formula 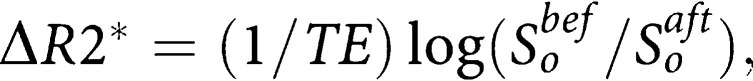 where TE is the echo time, and *S*_o_ the signal amplitude pre-USPIO (*S*_o_^bef^) and post-USPIO (*S*_o_^aft^), in the gradient-echo images. The same algorithm was employed to generate Δ*R*2 maps from the spin-echo images. For each mouse, after Δ*R*2^*^ and Δ*R*2 colormaps were generated, three slices were chosen, based on the maximum values of Δ*R*2^*^ in tumours, as assessed on the Δ*R*2^*^ colormaps. Δ*R*2^*^ and Δ*R*2 were measured in manually segmented ROIs on these slices. For each animal carrying Mel57-VEGF-A_165_ tumours, at least five lesions were analysed. In the animals carrying E34 and U87 tumours, multiple ROIs (*n*=9 per mouse) were defined within the tumour. Similarly, ROIs were defined within healthy brain regions (in total, nine ROIs in cortex and nine ROIs in striatum). For each ROI, mean Δ*R*2 and Δ*R*2^*^ were calculated by averaging the values of all pixels within the ROI. For each tumour line, as well as for healthy brain regions, the average and standard deviation was calculated from all ROIs. All algorithms were implemented in Matlab (Mathworks, Natick, MA, USA).

### Histological and immunohistochemical analysis

After the MRI experiments, animals were killed and the brains were removed and fixed in formalin. Coronal slices were embedded in paraffin and processed for immunohistochemical analysis. Vessels in E34, U87 and Mel57-VEGF-A_165_ lesions were highlighted by staining for the endothelial marker CD34 (Hycult Biotechnology, Uden, The Netherlands), glut-1 (Dakocytomation, Glostrup, Denmark; a marker for brain vasculature) and the pericyte marker *α*-smooth muscle actin (*α*-SM1; Sigma Chemical Co., Zwijndrecht, The Netherlands) according to previously published protocols (Kusters *et al*, 2002). To detect vascular permeability in the different tumours, sections were stained with HRPO-labelled anti-mouse IgG as described before (Kusters *et al*, 2002).

## RESULTS

**Figure 1:**
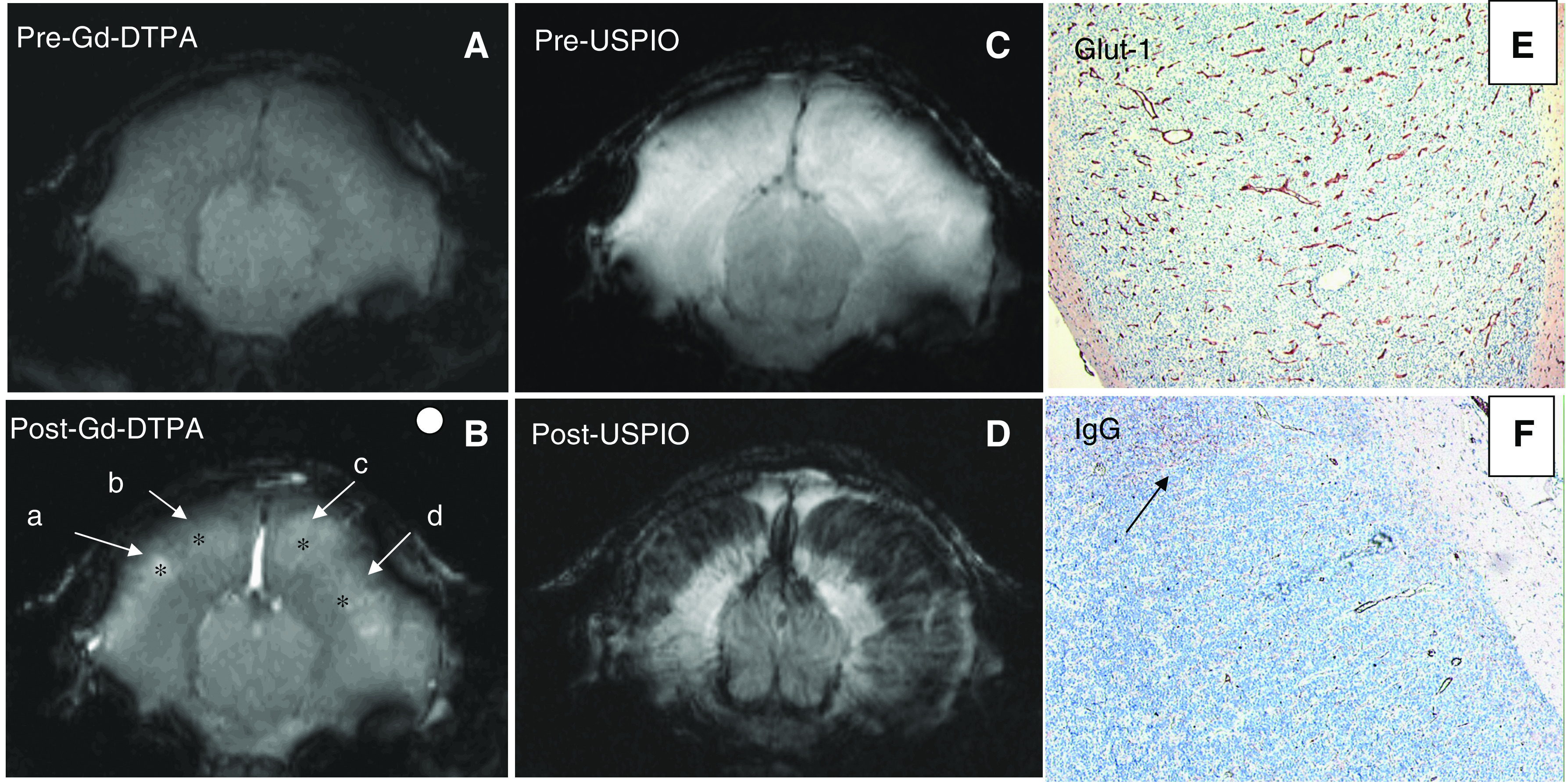
(**A**–**D**) Magnetic resonance images of mouse brain with a E34 glioma xenograft, acquired prior to (**A** and **C**) and following (**B** and **D**) administration of Gd-DTPA (**B**, gradient-echo images, TR/TE=400/6 ms, in-plane resolution=136 × 136 *μ*m) and USPIO (**D**, spin-echo images, TR/TE=2000/9 ms, in-plane resolution=136 × 136 *μ*m). In (**B**), the asteriscs (^*^) indicate the position of centre of the ROIs chosen for data analysis (see also Table 1) and the white circle indicates the size of the ROIs. (**E** and **F**) Immunohistochemical staining for glut-1 highlighting vessels (**E**) and immunostaining for extravasated mouse immunoglobulins (**F**). Note that vessel leakage is limited and heterogeneous. The arrow points at a tumour region where IgG has extravasated. Such regional leakage is in agreement with the heterogeneity in Gd-DTPA extravasation as shown in (**B)**. Note that the Gd-DTPA-enhancing region underestimates the extent of the glioma in both hemispheres (compare **B** and **D**).

**Figure 2:**
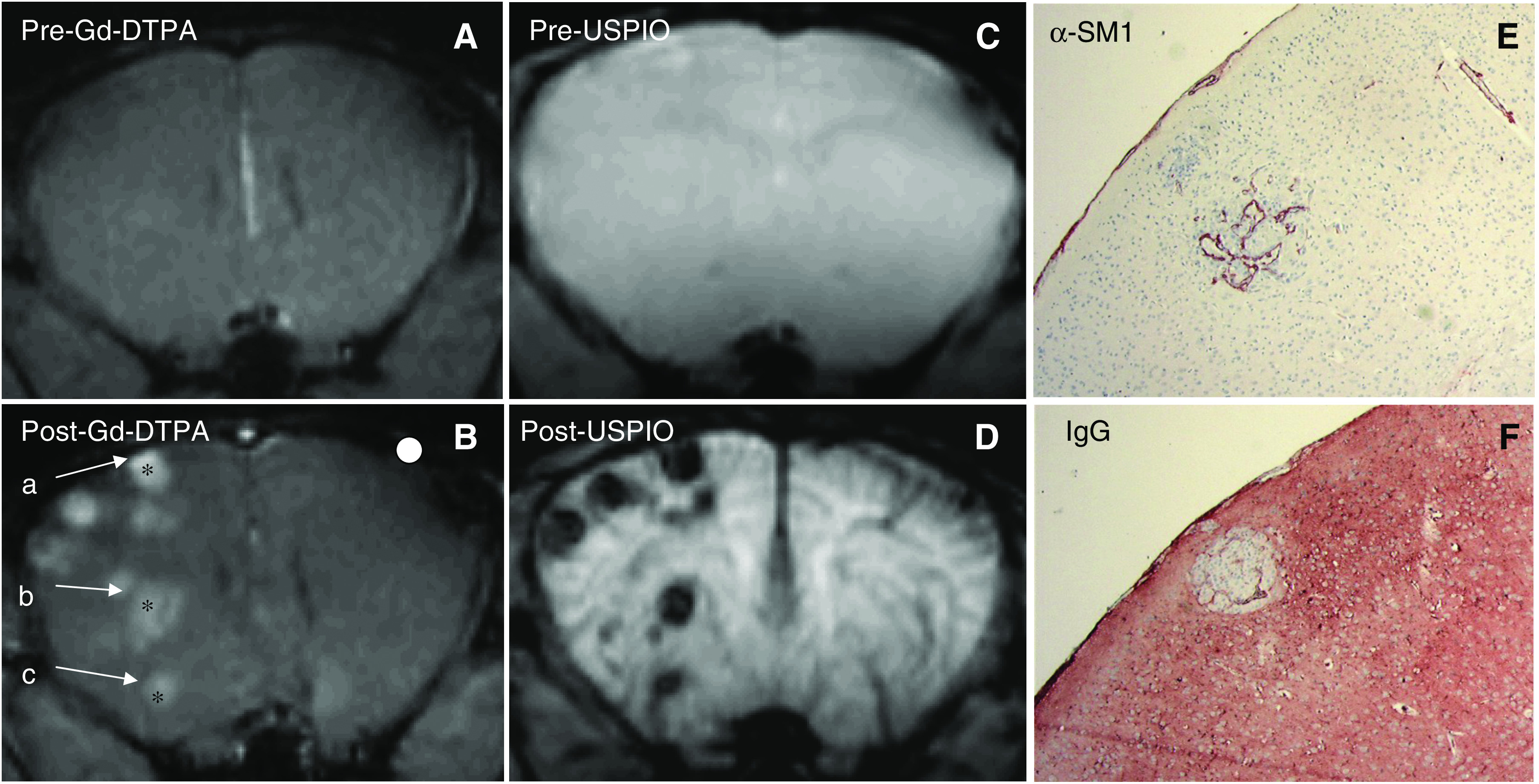
(**A**–**D**) Magnetic resonance images of mouse brain with Mel57-VEGF-A_165_ tumour, acquired prior to (**A** and **C**) and following administration of Gd-DTPA (**B**) and USPIO (**D**). The imaging parameters are the same as in Figure 1. From the image shown in the figure, three ROIs (labelled a, b and c) were analysed. (**E** and **F)** Immunostainings for pericytes (**E**) and extravasated IgGs (**F**) of a lesion in a section matched with the MRI slice. Note the highly dilatated tumour vasculature and the enormous amount of extravasated IgG.

**Figure 3:**
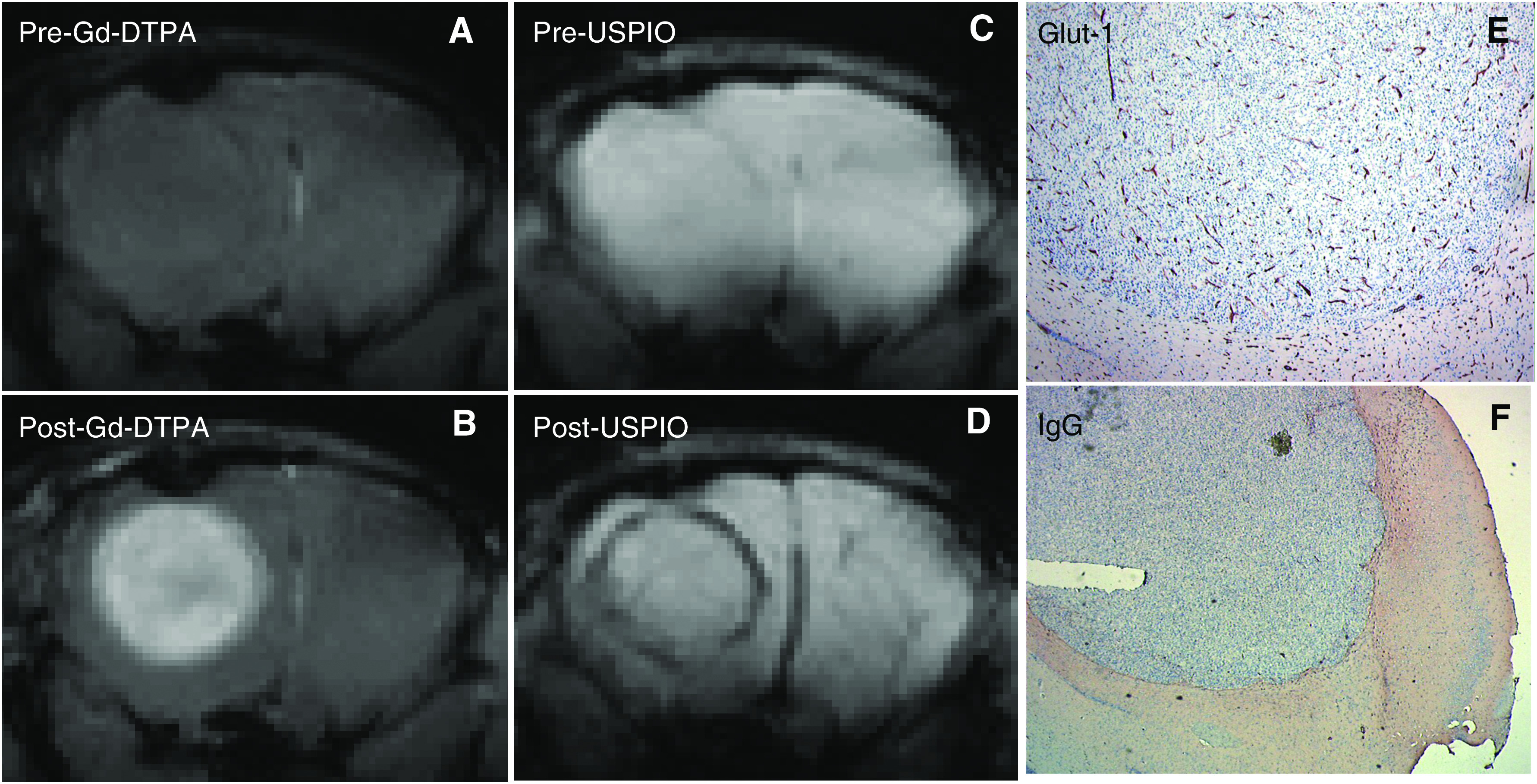
(**A**–**D**) Magnetic resonance images of mouse brain with a U87 tumour, acquired prior to (**A** and **C**) and following administration of Gd-DTPA (**B**) and USPIO (**D**), with the same imaging parameters as in Figure 1. (**E** and **F**) Immunohistochemical staining for glut-1 (**E**), highlighting vessels and an immunostaining for extravasated mouse immunoglobulins (**F**) confirm the contrast-enhanced MRI findings of high tumour blood volume and vessel leakage.

Precontrast MR images showed little or no contrast between tumour and healthy brain tissue in all three tumour models (Figures 1, 2 and 3A and C). Following Gd-DTPA and USPIO administration, tumours could be easily identified (Figures 1, 2 and 3B and D). Each tumour model displayed a distinctive pattern of vascular morphology and enhancement following administration of contrast agents.

**Figure 4:**
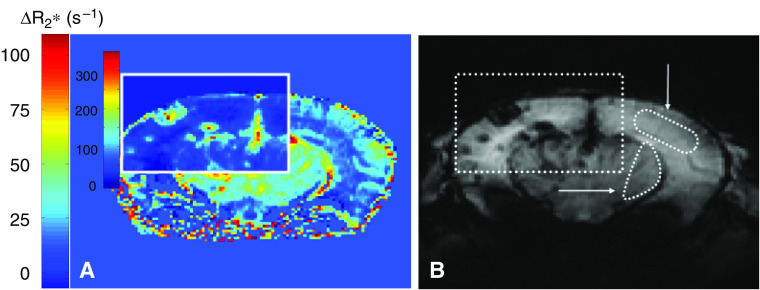
Pixel-by-pixel Δ*R*2^*^ map of murine brain with Mel57-VEGF-A_165_ metastases (**A**). Reference gradient-echo image post-USPIO is shown in (**B)**. The Δ*R*2^*^ map is displayed with a high cutoff threshold in the tumour areas (region inside the box) and a low cutoff threshold outside tumour regions, to better visualise the differences in regional blood volume between brain regions that do not include tumour. An example of two ROIs chosen for Δ*R*2^*^ and Δ*R*2 quantitation (see Table 1) in healthy brain regions – in the striatum (indicated by the horizontal arrow) and in the cortex (indicated by the vertical arrow) – is shown in the reference gradient-echo image.

Following Gd-DTPA administration, all tumours were hyperintense indicating Gd-DTPA extravasation. This correlated well with the vascular permeability in the tumours, as assessed by anti-IgG staining (compare panels F in Figures 1, 2 and 3). Following administration of USPIO, the E34 glioma showed high values of Δ*R*2 and Δ*R*2^*^ throughout the tumour (Figure 1D and Table 1). The Mel57-VEGF-A_165_ metastases appeared as black spots and showed an expansive growth pattern (Figure 2D–F), with very high values of Δ*R*2 and Δ*R*2^*^ (Table 1). Interestingly, this correlated well with the presence of highly dilated vessels (see *α*-SM1 staining in Figure 2E). The U87 tumours were characterised by a ring-like structure (Figure 3D), with relatively low Δ*R*2 and Δ*R*2^*^ values in the core and very high Δ*R*2 and Δ*R*2^*^ values in the peritumoral region. The values of Δ*R*2 and Δ*R*2^*^ were also measured in healthy brain regions (cortex: Δ*R*2=5±2 s^−1^ and Δ*R*2^*^=21±9 s^−1^; striatum: Δ*R*2=9±3 s^−1^ and Δ*R*2^*^=36±10 s^−1^). The macrovascular blood volume map of Mel57-VEGF-A_165_ tumours is shown in Figure 4A. In Figure 4B, the corresponding gradient-echo images are shown. A high threshold for Δ*R*2^*^ was used in the tumour areas (region inside the box). Outside the tumour region, a low cutoff threshold for Δ*R*2^*^ is used to maximise the contrast between striatum (ROI indicated by the horizontal arrow) and cortex (ROI indicated by the vertical arrow, Figure 4B). For clarity, these two ROIs are drawn only on the gradient-echo image.

## DISCUSSION

The ability to noninvasively detect aberrant vasculature is especially crucial for detection and delineation of brain tumours. In this study, we demonstrate that MR-based imaging of blood volume has important added value, as tumour vessels may be dilated in the absence of notable hyperpermeability, and hence may be better visualised by blood volume imaging. Specifically, in addition to qualitative assessment of vascular volume, which can be done by visually evaluating the change in signal intensity following USPIO, a quantitative assessment of blood volume can be performed by calculating Δ*R*2^*^. This allows for an objective comparison of vascular volumes of different tumours and also allows performance of longitudinal studies to assess, for example, treatment efficacy. An important part in the MR assessment of brain tumours in the clinic is the venous application of Gd-DTPA to detect blood–brain barrier leakage due to hyperpermeable tumour vessels. This results in interstitial Gd-DTPA accumulation, which can be vizualised on T1-weighted images. However, as brain tumours often show infiltrative growth without this leakage, no Gd-DTPA enhancement on T1-weighted MRI is then observed. In these cases, it is possible to use hyperintense lesions seen on T2-weighted MRI without contrast agent to characterise the tumour extent, but as oedema is also hyperintense on these images, tumour spread may be overestimated, and also underestimation by T2-weighted MRI has been reported (Pirzkall *et al*, 2001; Stadlbauer *et al*, 2004). In a few studies, it has been shown that T2^*^-weighted MR imaging after USPIO administration can have added value in the characterisation of human Gd-enhancing tumours.

In all tumour models used in this study, little contrast between healthy tissue and tumours was present in both T2- and T1-weighted images, with the specific acquisition timing used to obtain these images. Gadolinium-DTPA and USPIO contrast-enhanced MRI allowed not only detection of the tumours but also a more detailed characterisation of their vasculature. Overall, Mel57-VEGF-A_165_ tumours were characterised by higher blood volume than the E34 and U87 glioma xenografts. U87 tumours displayed higher blood volumes in the tumour rim than in the core. Again, we were able to confirm the MRI results with immunohistochemical stainings.

As Gd-DTPA and USPIO imaging were performed on the same mice, we were able to spatially correlate enhancement patterns of both contrast agents in the respective tumour models. It is well established that vascular volume and vascular permeability are among the best markers for *in vivo* tumour detection by MRI (Dennie *et al*, 1998; Su *et al*, 1998; Le Duc *et al*, 1999; Tropres *et al*, 2001; Turetschek *et al*, 2001; Verhoye *et al*, 2002; Bremer *et al*, 2003; Pathak *et al*, 2003; Robinson *et al*, 2003; van Laarhoven *et al*, 2006). It is generally assumed that tumours trigger angiogenesis or recruit blood vessels from the local vasculature, and, as a consequence, have increased local blood volumes. However, tumour cells may infiltrate healthy tissue and co-opt the pre-existing vasculature, a situation of particular importance in brain tumours (Kusters *et al*, 2002; Leenders *et al*, 2003). When this phenomenon occurs in the absence of angiogenesis or vascular alterations, this will result in a local decrease of blood volume, relative to surrounding healthy tissue. We previously showed that, upon USPIO administration, this decrease in blood volume translates into hyperintense voxels as compared to the surrounding tissue (Leenders *et al*, 2003). Thus, whether angiogenic or infiltrative, tumours have blood volumes that are distinct from the blood volume in adjacent normal tissue. These differences can be detected using blood-pool contrast agents such as USPIOs. Also, in light of our previous studies that showed that anti-VEGF treatment of brain tumours resulted in undetectability using Gd-DTPA MR imaging (due to restoration of the blood–brain barrier (Leenders *et al*, 2004; Claes *et al*, 2008)), USPIO imaging may be an attractive avenue to delineate brain tumours and evaluate antiangiogenic therapy.

For blood volume assessment of the macrovasculature and microvasculature, T2^*^-weighted gradient-echo and T2-weighted spin-echo images, respectively, need to be acquired prior to and following administration of USPIO. As shown in this study, the vascular morphology of the tumour is best visualised on post-USPIO gradient-echo images. Due to its high transverse relaxivity (∼100 mM^−1^ s^−1^, at 7 T (Gambarota *et al*, 2006)), USPIO provides an extremely high sensitivity to alterations in local tissue blood volume; as a result, it generates a high signal contrast in T2^*^-weighted images between regions of different blood volume. With respect to Δ*R*2, for typical USPIO concentrations employed in *in vivo* studies, only minor signal dephasing (and thus small Δ*R*2) originates in spin-echo images following USPIO administration (Le Duc *et al*, 1999; Gambarota *et al*, 2006). On the other hand, Δ*R*2 changes are sensitive to the small vasculature network, and therefore Δ*R*2 could be exploited for characterisation of the tumour microvasculature (Kennan *et al*, 1994; Weisskoff *et al*, 1994; Dennie *et al*, 1998; Le Duc *et al*, 1999). However, to detect USPIO-induced signal dephasing in spin-echo acquisitions, long echo times (∼100 ms) need to be employed, at the expense of the image SNR.

In the current study, all tumour models displayed vessel leakage, albeit at different degrees. Leakage of Gd-DTPA from tumour vessels into the interstitium is however not restricted to the tumour interstitium but can extent into the surrounding normal tissue. This obviously precludes an accurate estimation of tumour edges. As tumour (and edge) detection rely on factors that include (i) the signal intensity contrast between the lesion and peritumoral, non-neoplastic tissue and (ii) the spatial resolution of the MR image, USPIO (R2 relaxivity ∼100 mM^−1^ s^−1^) has the advantage over Gd-DTPA (R1 relaxivity ∼5 mM^−1^ s^−1^) of potentially improving both (i) and (ii).

In conclusion, detailed characterisation of the tumour vasculature by imaging vascular leakage and vascular volume provides a better understanding of the complex mechanisms associated with tumour development. Gadolinium-DTPA and USPIO contrast-enhanced MRI allow not only the detection of tumours but also characterisation of their vasculature, which is essential for diagnostic purposes and to assess efficacy of, for example, antiangiogenic therapy. Tumours from the three tumour models displayed three distinctive vascular morphologies, which were best assessed on post-USPIO gradient-echo images. The results of this study indicate that USPIO-induced Δ*R*2^*^ values are highly sensitive to small changes in regional blood volume. Thus USPIO imaging may be a very attractive alternative to Gd-DTPA imaging and will at least have added value, especially for detection and delineation of diffuse infiltrative brain tumours.

## Figures and Tables

**Table 1:** Mean and standard deviation of Δ*R*2 and Δ*R*2^*^

	Δ*R*2* (s^−1^)	**Δ*R*2 (s^−1^)**
Mel57-VEGF-A_165_	284±89	40±13
E34	153±36	17±5
U87 (rim)	54±15	13±5
U87 (core)	17±6	5±3
Cortex	21±9	5±2
Striatum	36±10	9±3

ROI=region of interest. Values of the mean and standard deviation of Δ*R*2 and Δ*R*2^*^in Mel57-VEGF-A_165_, E34 and U87 tumours and in healthy brain regions, averaged over all (ROIs).
